# Cancer Care Experiences Among Adolescents, Caregivers, and Health Care Providers in a Regional Canadian Context: Protocol for a Qualitative Study

**DOI:** 10.2196/76877

**Published:** 2025-09-15

**Authors:** Joanne Tay, Mohammad Jarrar, Telford Yeung, Jessica Kichler

**Affiliations:** 1 Faculty of Nursing University of Windsor Windsor, ON Canada; 2 Medical Oncology Windsor Regional Cancer Centre Windsor, ON Canada; 3 Department of Pediatrics Cumming School of Medicine University of Calgary Calgary, AB Canada; 4 Department of Psychology Faculty of Arts, Humanities and Social Sciences University of Windsor Windsor, ON Canada

**Keywords:** adolescents and young adults, cancer, caregivers, health providers, protocol, qualitative research

## Abstract

**Background:**

Adolescents diagnosed with cancer face significant disruptions to their development, education, and social lives. These challenges are pronounced in regional settings, where access to specialized, developmentally appropriate oncology care is limited. In Ontario, Canada, youth aged 12 to 18 years often fall between pediatric and adult care systems, leading to fragmented services, unmet psychosocial needs, and long-term disparities in survivorship. While international literature has examined the cancer experiences of adolescents and young adults, most research originates from the United States, limiting its relevance in the Canadian context. In Ontario’s regional hospitals, youth and families face disparities in care quality, specialist access, and logistical challenges. More research is needed to inform equitable, youth-centered cancer care models.

**Objective:**

This study aims to explore the lived experiences of youth cancer survivors, their caregivers, and health care providers (HCPs) in a regional Canadian context. The study investigates four research questions: (1) What are the daily experiences and psychosocial needs of youth during and after treatment? (2) How do caregivers navigate cancer care for youth? (3) What are HCPs’ perspectives on delivering cancer care for youth? (4) What recommendations can youth, caregivers, and HCPs offer to improve cancer care systems for youth?

**Methods:**

We applied a qualitative descriptive design using semistructured web-based interviews and reflexive thematic analysis. Participants were recruited through a multimethod strategy, including clinician referral, posters, digital outreach, and professional networks. The anticipated sample includes 24 participants: 8 (33%) youth cancer survivors (aged 12 to 18 years at diagnosis), 8 (33%) caregivers, and 8 (33%) HCPs. Eligibility criteria were defined to ensure safety, diversity, and relevance. Interviews were conducted via Microsoft Teams, transcribed verbatim, and analyzed using the 6-phase reflexive thematic analysis approach described by Braun and Clarke. NVivo software supported coding and theme development. Demographic data were analyzed descriptively to contextualize the findings.

**Results:**

As of September 2025, 14 participants had completed interviews: 6 (43%) caregivers, 6 (43%) HCPs, and 2 (14%) youths. Youth recruitment has been challenging due to the developmental stage and competing commitments. Data collection concluded in December 2024. Preliminary transcript coding was completed in early 2025, with final analysis and synthesis of themes completed in June 2025. This study was funded in August 2023, and results are expected to be published in Fall 2025 and Winter 2026.

**Conclusions:**

This study will provide critical insight into cancer care delivery for youth in a regional Canadian setting. Integrating youth, caregiver, and HCP perspectives will illuminate systemic gaps, relational dynamics, and context-specific barriers. The findings will inform policy, education, and service innovations aimed at improving equity, continuity, and developmental responsiveness in oncology care for adolescents and young adults in Canada.

**International Registered Report Identifier (IRRID):**

RR1-10.2196/76877

## Introduction

### Impact of Cancer on Youth

The onset of cancer at any age can have a devastating impact on a person’s physical and mental health. This experience may be especially distressing for youth, who are establishing their identity, values, and autonomy while also learning to navigate relationships and life transitions during adolescence [1]. Studies have shown that youth often perceive cancer treatment as emotionally and psychologically challenging, as they confront uncertainty and the possibility of death [1-3]. Many report feelings of isolation and hopelessness, which contribute to difficulties adhering to treatment plans and delays in accessing appropriate supportive care [4]. During treatment, some youth may continue attending school. However, cancer and its treatments can impair cognitive function, memory, abstract reasoning, and arithmetic skills, making reintegration into the classroom environment challenging [5]. Socially, these youth may experience differential treatment from peers, which can further exacerbate their sense of isolation [1,2].

The transition to young adulthood is often an exciting and pivotal time [6]. However, for youth diagnosed with cancer, this period may be disrupted by health adversities. A cancer diagnosis can delay their ability to complete education, secure employment, and form new relationships, setting them back in their developmental trajectory [1,7]. Research also indicated that adult survivors of childhood cancer face long-term physical and mental health complications [1,8,9]. These issues may be compounded by complex socioenvironmental factors, such as low socioeconomic status, geographic isolation, and limited social and community support [10]; for instance, inadequate communication between health care providers (HCPs) and schools regarding accommodations for youth undergoing treatment for cancer reflects a systemic gap that may hinder educational continuity and psychosocial development [5,11]. Adult survivors also report long-term difficulties in employment, intimate relationships, and social participation, issues that often begin during treatment and persist throughout survivorship [12]. Providing age-appropriate, integrated clinical and psychosocial care is crucial for mitigating these challenges. However, in the Canadian province of Ontario, oncology care is distinctly divided into pediatric and adult systems, failing to address the unique needs of adolescents and young adults adequately [13]. These youth may be overlooked without early intervention and tailored support.

### Existing Research on Cancer Care for Adolescents and Young Adults

The majority of research examining the cancer experiences of adolescents and young adults (AYAs) has been conducted in the United States, where studies have identified unique challenges faced by this population that differ from those of both pediatric and adult patients with cancer [11,14-16]. While both American and Canadian families face challenges related to geographic access and wait times, these issues are shaped by distinct health care structures. In the United States, barriers often vary by insurance status, while in Canada, they stem from regional resource distribution within a publicly funded system [13,17,18]. Furthermore, US research frequently focuses on resourcing urban tertiary care centers with specialized AYA programs [17,19,20]. By contrast, the Canadian health care landscape is characterized by a more distributed model of regional care delivery, and structural differences in educational support frameworks may influence how findings translate across contexts [21-23].

### Disparities in the Regional Health System That Impact Youth With Cancer

Regional hospitals in Ontario are designed to deliver centralized care to their local populations [13,23]. However, studies highlight significant disparities in the availability and quality of cancer care between tertiary and regional treatment centers [24]. Youth receiving care in regional centers may have access to some outpatient and routine oncology services. However, these facilities often cannot manage youth with acute or complex medical conditions. As a result, families must travel outside their communities to access essential care, disrupting social support systems and imposing emotional, logistical, and financial burdens that can negatively affect overall quality of life [25]. These disparities underscore the need for more equitable and developmentally appropriate care across regions. Adolescents have needs that are distinct from those of children and adults; yet, these needs often remain unaddressed due to a lack of awareness, guidelines, and available resources [7,9].

Regional pediatric oncology clinics in Ontario, supported by the Pediatric Oncology Group of Ontario, primarily offer supportive and clinical care to patients up to 18 years of age [26]. However, for initial diagnoses or the management of complications, youth and their families must travel to tertiary centers [11,24,27]. The need to travel outside their communities to seek care can be particularly stressful due to unexpected costs, lost work hours for parents, and separation from family and community supports [10,17,28]. These challenges are often amplified for youth from single-parent households, immigrant families, or low-income backgrounds, where access to social supports is limited, contributing to long-term mental health consequences [1,24].

A literature review on the impact of travel on cancer treatment decisions highlighted that travel is perceived as a barrier to treatment adherence [25]. Transportation mode, parking access, reliance on hospital transport, and travel comfort all influence the overall cancer care experience [25]. However, most studies to date have focused on adult populations; little is known about how these travel-related barriers affect youth with cancer living far from tertiary centers, who may rely on others for transportation. These systemic challenges also place a burden on HCPs in regional settings, who are often the first point of contact for these patients [10,27]. Studies reveal that HCPs experience challenges in identifying and addressing the emotional and practical needs of youth with cancer and their families [18,29,30]. Youth may present with symptoms and psychosocial challenges that differ from those of younger children or adults, making specialized training essential. A lack of training can lead to delays in care and unmet needs [7,31,32]. While referral to tertiary centers may provide access to specialist care, enhancing training and resources in regional and community hospitals is critical [13]. Building local capacity through targeted education and support for HCPs is necessary to ensure timely, effective, and youth-appropriate cancer care closer to home [1,30].

### Context Specific to Southwestern Ontario

Ontario’s pediatric oncology care operates under a hub-and-spoke model where specialized pediatric oncology services are centralized in major urban centers (Toronto, Ottawa, Hamilton, and London), with regional satellite clinics such as Windsor Regional Hospital (WRH) providing supportive care closer to home [23]. This structure can complicate transitions between pediatric and adult services, especially for adolescents with complex needs [33,34]. The Windsor-Essex region, serving approximately 422,000 residents with significant immigrant populations and diverse socioeconomic backgrounds, faces additional complexities as a border community with unique demographic characteristics and economic patterns tied to the automotive manufacturing industry [35]. In Windsor-Essex, youth experience longer wait times for mental health care and limited access to specialized AYA supports, adding to the aforementioned challenges [21,33].

### Research Gap in the Canadian Context

No published Canadian studies have specifically examined the lived experiences of adolescent patients with cancer and their families within regional health care settings. While Canadian cancer surveillance data provide epidemiological insights [26], there is a lack of qualitative research exploring how the unique features of Canada’s regional care delivery shape the cancer experience for young people and their families. This gap is especially significant, given that approximately 40% of Canadian youth live in nonmetropolitan areas, where regional hospitals serve as their primary points of care [15,26]. This study was developed in response to these gaps and informed by guidance from the funding agency (Multimedia Appendix 1).

### Study Objectives

Given the research gap in Canadian context–specific AYA cancer research and the unique health care delivery challenges faced by youth in regional settings such as Windsor-Essex, this study addresses important knowledge needs that cannot be met through extrapolation from US-based literature. This study examines the impact of Ontario’s regional cancer care system and health care structure on the experiences of adolescents, caregivers, and HCPs. The research questions guiding this study are as follows:

1. What are the everyday experiences and psychosocial and support needs of youth during and after routine cancer treatment?
2. How do parents and caregivers navigate the process of receiving routine cancer treatment with their youth?
3. What are the perspectives of HCPs about youth receiving routine cancer treatment?
4. What recommendations do the youth, parents and caregivers, and HCPs have for relevant decision-makers and partners (eg, community organizations, government and policymakers, and hospital administration) to improve cancer care for youth?

## Methods

### Study Design

This study used a qualitative descriptive approach, guided by the reflexive thematic analysis approach described by Braun and Clarke [36], to explore the lived experiences, care processes, and recommendations related to cancer treatment among youth, caregivers, and HCPs in Windsor-Essex Region. This protocol and subsequent reporting adhere to the COREQ (Consolidated Criteria for Reporting Qualitative Research) guidelines, ensuring comprehensive and transparent reporting of the qualitative research methodology and findings [37]. While much of the existing research on the cancer experiences of youth originates in the United States, contextual differences in health, education, and social systems necessitate Canada-specific inquiry [1,38]. Guided by the four research questions outlined in the Study Objectives subsection, this study focused on (1) the day-to-day experiences and psychosocial needs of youth during and after cancer treatment, (2) the caregiving and care navigation experiences of parents and caregivers, (3) the perspectives of HCPs, and (4) key member–informed recommendations for improving care. Data were collected through web-based semistructured interviews and analyzed using inductive thematic analysis.

### Participant Demographics

This study was conducted in Southwestern Ontario and focused on youth receiving regional oncology care. The sample size of 8 participants per group was determined based on qualitative research guidelines, which suggest that thematic saturation typically occurs within 6 to 12 interviews per homogeneous group [39]. Given the perspectives of 3 distinct key members (youth, caregivers, and HCPs) representing different experiential domains, we anticipated that 8 participants per group would provide sufficient data richness within each stakeholder category. A purposive sampling strategy was planned to ensure diversity in participant experiences and demographics. Recruitment actively sought participants from different racial and ethnic backgrounds, including the region’s Arab, South Asian, and other immigrant communities, with varying socioeconomic statuses, cancer diagnoses (including both hematologic and solid tumor malignancies), and treatment experiences. Recruitment materials were distributed through culturally diverse community organizations, recognizing that the English fluency requirement may limit participation from some non–English-speaking families.

Eligible youth participants included those diagnosed with cancer between January 2014 and December 2023 and who were aged between 12 and 18 years at the time of diagnosis. Youth must have received part or all of their oncology care at a regional hospital and demonstrate sufficient cognitive and communicative ability to consent and participate in an interview independently. Primary caregivers of eligible youth will also be invited to take part in the study.

HCPs, such as oncologists, nurses, therapists, or social workers, were eligible if they were currently employed at regional hospitals and provided care to youth with cancer and their families. As noted earlier, participation was limited to English-speaking individuals due to resource constraints, which may affect the diversity of the sample. Youth were excluded if they were diagnosed with cancer within the 3 months before study enrollment or if the referring pediatric oncologist determined that participation may be psychologically harmful. This may include cases in which the youth’s cancer experience was traumatic, and participation could trigger significant mental health concerns. This sampling approach ensures that participants are prepared to reflect meaningfully on their experiences in a supportive research environment.

### Recruitment

A multimethod sampling strategy was used to recruit youth cancer survivors, their parents or caregivers, and HCPs involved in their care. Recruitment occurred through 4 different means to support broad accessibility, minimize selection bias, and enhance participant diversity. The main recruitment involved clinician-facilitated referral at the regional hospital satellite oncology clinic. During routine outpatient visits, eligible youth and caregivers were introduced to the study by their oncology clinician using a standardized study information letter. This letter provided an overview of the study’s objectives, procedures, and ethical safeguards, and included a QR code linking to a secure REDCap (Research Electronic Data Capture; Vanderbilt University) screening form. Interested individuals completed the form independently at their convenience using a personal device. To protect voluntariness and reduce the potential for perceived coercion, clinicians were not informed of who proceeded with enrollment.

To complement this approach, recruitment posters were displayed throughout clinical settings, including waiting rooms, outpatient areas, and staff bulletin boards. Posters were tailored to each participant group, such as youth, caregivers, and HCPs, and included a QR code linking to the REDCap screening form. This passive recruitment strategy allowed potential participants to self-identify and engage with the study without direct clinician involvement. Digital recruitment was conducted through the website of the Pediatric Oncology Group of Ontario and the research team’s social media channels, including Twitter (subsequently rebranded as X) and Instagram, to further broaden outreach beyond the clinical environment. Electronic flyers were designed specifically for youth and caregivers and contained a study email address and a QR code directing those interested to the REDCap screening form. This digital strategy was intended to reach individuals who may no longer be in regular contact with oncology care services, thereby improving accessibility.

In parallel, HCPs were recruited through email invitations distributed by clinical managers and department heads at a regional hospital. These emails contained a study summary, assurances regarding voluntariness and confidentiality, and a link to the REDCap screening form. Posters were also displayed in staff common areas to reinforce recruitment messages. While clinical leaders promoted the study broadly, they will not be involved in identifying or enrolling individual participants. This ensures a clear separation between research and professional roles.

Once a screening form was submitted, the research assistant reviewed responses to determine eligibility based on predefined inclusion and exclusion criteria. Eligible participants were then contacted, provided with the study letter of information, given the opportunity to ask questions, and invited to schedule a web-based consent appointment. A follow-up email was sent if no response was received within 2 business days. Individuals deemed ineligible received a standardized email indicating that they did not meet the study criteria. The research team independently conducted all consent and enrollment procedures to uphold ethical standards and maintain participant confidentiality.

### Data Collection

Data were collected through interviews using Microsoft Teams, a secure videoconferencing platform licensed and supported by the University of Windsor. Before the interview, we completed a brief demographic questionnaire with all participants via REDCap to gather descriptive information, including age, gender identity, location, relationship to the youth (for caregivers), and clinical role (for HCPs). These data will help characterize the sample and provide important context for interpreting the qualitative findings [40].

The research team developed the interview guides (Textbox 1) for each participant group (youth, parents, and HCPs), drawing from existing literature and the study’s research questions. Draft guides were reviewed by the research team (JT and JCK) and assessed to ensure that questions were clear and sensitively worded. Revisions were made based on this feedback before finalizing the guide. The research team piloted the guide to ensure clarity, relevance, and sensitivity to participant experiences [41]. Questions were open-ended and conversational, encouraging participants to reflect on their experiences in their own words. Youth interviews focused on daily life, emotional well-being, and transitional challenges experienced during and after treatment. Caregiver interviews focused on the emotional impact of caregiving, experiences with accessing and navigating services, and care coordination responsibilities. HCP interviews examined routine cancer care delivery, interdisciplinary collaboration, systemic barriers, and recommendations for improving care for youth [1,42].

**Interview guides for youth, parents or caregivers, and health care providers.**

**Interview guide for parents or caregivers and youth diagnosed with cancer**

1. Can you tell me a little bit about yourself and your family?
2. What has it been like for you and your child to live with cancer?
3. Can you tell me about the resources that you used or were referred to during your cancer treatment journey? In what ways did you find them helpful or not helpful?
4. What kinds of things do you and your parent (or you and your child) talk about when it comes to the cancer experience?
5. Can you tell me about how your health care providers prepared you (or your child) for treatment and what to expect afterward?
6. Can you tell me a bit about what is most important to you in life right now?
7. What do you hope to see improved in terms of cancer care for youth living in Southwestern Ontario?
8. Is there anything else you feel that we did not cover today or that you feel is important for me to know about this topic?

**Interview guide for health care providers**

1. Can you tell me a little bit about yourself and your role as a (health care provider, eg, oncologist, dietitian, registered nurse, or occupational therapist) in the oncology satellite unit?
2. What experience do you have providing oncology care specifically to the adolescent and young adult population?
3. Could you describe the kinds of community and hospital or clinic resources that are available in Southwestern Ontario for youth diagnosed with cancer and their families?
4. What do you think are some of the important considerations that youth have in mind when undergoing their cancer treatment?
5. How do you think cancer care in the oncology satellite unit at the regional hospital can be improved for youth with cancer?
6. Is there anything else you feel that we did not cover today or that you feel is important for me to know about this topic?

Each interview was scheduled at a time convenient for the participant and lasted approximately 1 hour. Participants had the option to pause or take breaks at any time. The research assistant was available to coordinate a follow-up session for those choosing to complete their interview in 2 parts. Verbal reconfirmation of consent was obtained before resuming a second session. The principal investigator, coinvestigator, or a trained research assistant conducted interviews. To support participant comfort and ensure confidentiality, interviewers were not individuals currently or formerly involved in the participants’ clinical care.

All interviews were audio recorded with participant consent, transcribed verbatim, and deidentified during transcription. The research team reviewed transcripts for accuracy. Once transcription was complete, audio files were permanently deleted. All interview procedures and communications were designed with flexibility and sensitivity to accommodate varying levels of comfort and emotional readiness, particularly given the potentially distressing nature of reflecting on cancer-related experiences [1,42]. A list of mental health and community resources was provided at the time of consent and again after the interview to ensure that participants were supported throughout the process.

### Data Analysis Plan

Interview data are being analyzed using reflexive thematic analysis, following the 6-phase methodology outlined by Braun and Clarke [36]. This approach is consistent with the study’s qualitative descriptive design and pragmatic orientation, allowing for a systematic exploration of how youth, caregivers, and HCPs experience and make meaning of routine cancer care [40]. The study is situated within a relativist ontology and constructionist epistemology, recognizing that participants’ realities are subjectively constructed through their lived experiences [43]. This philosophical positioning aligns with the study’s goal of capturing diverse perspectives and meaning-making processes across participant groups. The research team transcribed all interview recordings verbatim, deidentified them, and verified their accuracy before coding. The transcripts were then imported into NVivo 14 (Lumivero), which is being used to support data management, code organization, and theme development.

The analytic process is iterative and reflexive, guided by the study’s 4 core research questions. Analysis begins with open coding, allowing for the inductive identification of meaningful data across transcripts. Codes are grouped into categories and abstracted into themes across participant groups [40,41]. Multiple research team members are involved in coding, theme generation, and theme refinement to enhance analytic rigor. Regular team meetings are held to compare interpretations, resolve discrepancies, and ensure consistency in analytic decision-making.

Thematic saturation is assessed using an iterative approach during concurrent data collection and analysis. Saturation is considered achieved when (1) no new codes emerge from consecutive interviews within each participant group, (2) existing themes are well developed with sufficient depth and variation, and (3) additional interviews yield redundant information that does not enhance thematic understanding. The research team meets after every 6 interviews within each group to assess progress toward saturation. If the eighth participant in any group does not achieve saturation, recruitment will continue with up to 2 additional participants per group until saturation is reached. Given the recognized challenges in recruiting youth participants due to their developmental stage and competing life priorities, the assessment of saturation for the youth group is evaluated with particular attention to the depth and richness of available data rather than strictly adhering to numerical targets. If youth data do not meet saturation criteria, this limitation will be acknowledged and the available data analyzed for depth and contextualized with caregiver and HCP perspectives. The final sample size will be determined by data adequacy rather than predetermined numbers.

To establish credibility, debriefing occurs within the research team throughout the analytic process. Transferability is addressed by providing a rich description of participant contexts and characteristics, enabling readers to assess the applicability of the findings to other settings [44]. Trustworthiness is established through the use of participant quotes for each theme, demonstrating how the original data support the interpretations. Dependability is ensured by documenting all analytic decisions, including coding frameworks and iterative changes to themes. An audit trail is maintained to establish confirmability, linking thematic interpretations to original data excerpts and providing transparency in the analytic process, as positioned by each research team member [45].

To further contextualize the findings, thematic comparisons are made between youth, caregiver, and HCP interviews to identify convergence, divergence, and potential gaps in perspectives across these groups. This synthesis supports the generation of nuanced insights and informs evidence-based recommendations for improving cancer care for youth in the Canadian context [1,42]. Quantitative demographic data are analyzed using descriptive statistics (eg, means, SDs, and frequency distributions) to characterize the sample. These findings will be presented alongside the qualitative results to provide context for the participant narratives in the manuscripts.

### Ethical Considerations

This study has received approval from the University of Windsor Research Ethics Board (24-027). Informed consent was obtained electronically using REDCap after a clear explanation of the study was provided. Participants were assigned a study ID to maintain confidentiality, and all data were deidentified and stored securely in password-protected OneDrive (Microsoft Corporation) folders at the University of Windsor. The research team adhered to the Personal Health Information Protection Act and institutional policies to ensure participant confidentiality. Audio recordings were securely deleted after transcription and analysis, and all deidentified study data will be destroyed after 7 years. The anticipated risk of participation was assessed as low to moderate. Some youth and caregivers may have experienced emotional discomfort during interviews due to reflections on illness experiences. The principal investigator, a registered pediatric nurse, trained research assistants to identify signs of distress. If distress occurred, interviews were to be paused, and participants were to be offered support or referred to psychosocial services. The coprincipal investigator, a licensed clinical and health psychologist, was available for urgent consultation and triaging, if necessary. All participants were provided with a mental health resource sheet. Participation was voluntary and did not influence the clinical care that participants received. Each participant received a CAD $30 (approximately US $21.70) electronic gift card via email after their interview, regardless of whether it occurred in one or multiple sessions. Participants were able to withdraw at any time during the interview. Withdrawal after interview completion is not possible due to subsequent deidentification of the data and analyses of the data in aggregate. Participants who withdrew during an interview were still eligible to receive the full compensation for their participation.

## Results

Participant recruitment commenced in November 2023 after ethics approval was obtained. As of September 2025, a total of 14 participants had completed semistructured interviews, including 6 (43%) caregivers, 6 (43%) HCPs, and 2 (14%) youths. Youth recruitment has been limited, likely due to their current life stage, as many are in remission and balancing employment or postsecondary education. Follow-up outreach efforts yielded minimal responses. The final achieved sample represents 58% (14/24) of the planned recruitment target, with caregiver and HCP recruitment reaching 75% (6/8) of their respective targets, while youth recruitment achieved only 25% (2/8) of the intended sample. Interview transcription was conducted between January and March 2025, with all interviews ranging from 45 to 75 minutes. Currently, preliminary analysis is in progress, with 2 research team members having independently coded initial transcripts and met to compare and refine coding. Initial coding has revealed themes related to care coordination challenges, psychosocial support gaps, and systemic barriers within regional cancer care delivery, although comprehensive thematic development awaits completion of the full analytic process. Coding and theme generation were finalized in June 2025.

## Discussion

### Expected Contributions

This study seeks to generate contextually rich insights into the lived experiences of youth cancer survivors, their caregivers, and HCPs in a regional Canadian setting. While most US research has focused on challenges such as insurance issues, treatment costs, and access to specialized AYA programs in large urban tertiary centers, this study examines how Canada’s public health care system and regional care delivery models shape the cancer experience for adolescents and their families. Using reflective thematic analysis, we explore how youth navigate the psychosocial, emotional, and practical challenges of cancer care; how caregivers manage care coordination; and how HCPs address the unique needs of this population. This multiperspective approach brings together different experiences to better understand how the overall care journey is shaped by relationships; systemic barriers; and local factors such as health care infrastructure, geographic access, and social support. The findings may highlight service gaps and suggest ways to improve continuity, developmental responsiveness, and equity in cancer care. They will also contribute evidence to support capacity building in regional settings and guide local and provincial planning. This work fills a gap in Canadian research and supports ongoing improvements in care delivery and health system design, building on insights from participants’ lived experiences.

### Dissemination Plans

The findings from this study will be disseminated to engage knowledge users. Study results will be shared with youth, caregivers, clinicians, administrators, and policymakers through multiple formats tailored to their preferences. Dissemination activities include academic publications and presentations at provincial and national oncology conferences.

### Future Directions

This protocol lays the groundwork for several future research initiatives. First, insights from this study will inform the design of a larger, multisite study examining cancer care for youth across different provinces and health system models in Canada. Second, the findings may be used to codevelop and pilot an intervention targeting identified gaps, such as youth-centered survivorship planning or caregiver navigation support. Given the anticipated identification of system-level barriers and training needs among HCPs, future work may also focus on cocreating and evaluating continuing education modules to enhance provider competence in youth-centered oncology care, especially in regional settings. In addition, longitudinal follow-up with youth participants could explore how their experiences evolve during the transition to adulthood, including re-engagement with the health care system, vocational development, and long-term psychosocial adjustment.

### Strengths and Limitations

A key strength of this study is its focus on a population often underrepresented in oncology research: youth in regional settings who must navigate complex care pathways with limited support. The use of a qualitative descriptive design and reflexive thematic analysis, grounded in a pragmatic orientation, allows for rich, context-sensitive insights to emerge directly from participants’ narratives. The inclusion of multiple perspectives (youth, caregivers, and HCPs) enables the triangulation of findings and highlights systemic patterns and relational dynamics that would otherwise remain invisible. Web-based interviews increase accessibility and flexibility for participants, which is particularly important for those balancing work, school, and health-related appointments.

However, there are also limitations. Recruitment challenges among youth in remission may limit the impact of youth perspectives beyond the current subgroup that is represented in this study. The reliance on a single regional setting may reduce transferability to other geographic contexts with different health system configurations. In addition, while efforts were made to ensure diversity in participants’ sociodemographic backgrounds, those with limited internet access or English fluency may have been unintentionally excluded. Finally, despite efforts to take the research team’s positioning into account through reflexive analysis and team-based coding, interpretation remained subject to the perspectives and lens of the research team [45].

### Conclusions

Adolescents with cancer face unique and often overlooked challenges as they navigate treatment, schooling, social transitions, and their emerging identities. These challenges are often compounded for youth living in regional settings, where access to developmentally appropriate and specialized oncology care is often limited. Despite growing international interest in AYA oncology, there remains a critical gap in Canadian, context-specific research that captures the lived experiences of youth, their caregivers, and the HCPs who support them. This study offers in-depth insights into how youth and families experience cancer care in a regional Canadian setting, identifying key factors that influence equity and continuity. By examining multiple perspectives and centering the perspectives of youth, this work will potentially inform future research, practice, and policy development aimed at improving equity and responsiveness in cancer care for adolescents. Through its focus on local context, collaborative dissemination, and implications for broader system change, the study lays the foundation for scalable improvements that better reflect the realities of youth navigating cancer within the Canadian health care system.

## Appendix

Multimedia Appendix 1 Peer-review report by Multidisciplinary Research Assessment Committee, WE-SPARK Igniting Discovery Grant Program.

## Abbreviations

AYA: Adolescent and young adult
COREQ: Consolidated Criteria for Reporting Qualitative Research
HCP: health care provider
REDCap: Research Electronic Data Capture

## Data Availability

The datasets generated or analyzed during this study are available from the corresponding author on reasonable request.
