# Supplementary material for: Cancer Care Experiences Among Adolescents, Caregivers, and Health Care Providers in a Regional Canadian Context: Protocol for a Qualitative Study
Source: JMIR Res Protoc. 2025 Sep 15;14:e76877. doi: 10.2196/76877 (PMC12481136; doi:10.2196/76877)
Supplement: Multimedia Appendix 1 [file resprot_v14i1e76877_app1.pdf]

Igniting Discovery Grants Program
2023 Competition
Scientific Officer Comments

Your grant was reviewed by the Multidisciplinary Research Assessment Committee made up primarily of external reviewers, a Scientific Officer and the Committee Chair**.** Detailed feedback provided by the Reviewers are attached. The comments below indicate the consensus score and additional discussion points that may not be included in individual reviewers' comments. If you have any questions, please contact us: wesparkhealth@uwindsor.ca

**Surname, Given name:**	Tay, Joanne	**Institution:**	University of Windsor
**Faculty/ Department:**	Nursing	**Email:**	jtay@uwindsor.ca

	CONSENSUS	
Team	4.00	Outstanding
Proposal/Merit	2.00	Strong
Impact	3.00	Very Strong
Weighted Score	2.75	Very Strong

Project Title: Exploring the process of receiving pediatric cancer care in Windsor Essex Region through the lived experiences of youths living with cancer, parents, and healthcare providers.

There was consensus across reviewers that this is a unique proposal for the region and if successful, would support an early career researcher build their research program.

There was some discussion around the merit of the proposal. Some suggestions were made regarding sampling and recruitment, e.g., will the sampling matrix work with only 8 youth as the target sample? May want to consider using translation services for non-English speaking participants. Consider recruiting people currently on treatment to make it easier to connect with potential participants. Additional details are needed regarding data collection and analysis.

One reviewer thought the research questions could have been more specific, less focused on program evaluation and framed around the specific expertise of the researcher. This would have increased their score on impact.

Reviewers were impressed with the focus on a knowledge translation activity to communicate findings to the community. It is a tangible opportunity to showcase their findings in a way that other proposals did not.

Reviewers were in agreement that this project has the potential to lead to larger funding. Please refer to individual reviewer's comments for details.

WE-SPARK Igniting Discovery Grant Program
2023 Competition
Reviewers Comments

Project Title: Exploring the process of receiving pediatric cancer care in Windsor Essex Region through the lived experiences of youths living with cancer, parents, and healthcare providers.

Brief Overview of Proposal. This study: 1) explores the experience of youths who were diagnosed with cancer and received care at Windsor Regional Hospital (WRH), 2) explores parents'/caregivers' perceptions about their child's cancer diagnosis and the support they received from WRH, 3) examines the perspectives of HCPs on caring for youths with cancer at WRH, and 4) identify gaps in areas of medical and psychosocial care that impact youths' experience and quality of life. Some rationale for the study is based on a specific need for Canadian-based data.

Strengths and Weaknesses Noted Within Each Category on the Reviewer Grid.

Excellence of Researcher/Team:

- Team includes both strong and necessary research and clinical team members with content and methods expertise. The PI and other team members have undertaken EDI training and engaged in other research focused on equity-seeking populations.

Merit of the Proposal:

- The proposal is well written with a strong background and rationale for the proposed work. The methods include a clear commitment to diversity in participants within specified groups (e.g., family composition, gender identity). Participant inclusion criteria is clearly outlined and planned content analysis process described. Strength of the proposal to see a knowledge translation plan.
- Some necessary details are missing from the proposal, including number of participants to be recruited, as well as feasibility of the participant recruitment strategy (i.e., how many participants are seen at WRH? is it feasible to recruit the diverse sample as described?). The research team should consider using available qualitative analysis software (NVivo) over Microsoft Word. With some important missing details, it is difficult to assess feasibility of the project within a 1-year timeframe.
- Part of the rationale for the project is that "there are fundamental differences in the social-environmental systems between [Canada and the US]"; however, the researchers don not go into much detail as to what those important differences are and how those are specifically relevant to their population and research questions. This is important to clarify as data is only being collected at one site (WRH) vs across Canada, thereby limiting its generalizability across the country.
- The background information speaks specifically about transition from pediatric to adult care, as well as coordinating care between tertiary and regional care centers. The research proposal would be strengthened with a stronger emphasis on these key priorities areas within the

research questions themselves. This will help to clearly elevate the unique contributions of this specific project.

- Some discrepancies in the budget (e.g., research staff lists need for 18% benefits, but then budget calculated on 14% benefits, 30 trips for RA travel but then only budget for 20)

Impact:

- A strength to see the research team budget for an end-of-grant forum to share findings back with the community. They may consider opening this up to a broader community beyond participants as an opportunity for broader engagement and impact with their knowledge mobilization to various audiences.

WE-SPARK Igniting Discovery Grant Program
2023 Competition
Reviewers Comments

Project Title: Exploring the process of receiving pediatric cancer care in Windsor Essex Region through the lived experiences of youths living with cancer, parents, and healthcare providers.

Brief Overview of Proposal.

This proposal is for a qualitative descriptive study designed to answer the following research questions in the context of youth cancer care in the Windsor-Essex Region : (1) What are the everyday experiences and psychosocial and support needs of youth during and after routine cancer treatment?; (2) How do parents/caregivers navigate the process of receiving routine cancer treatment with their youth?; (3) What are the perspectives of HCPs about youth receiving routine cancer treatment? (4) What recommendations do the youths, parents/caregivers, HCPs have for relevant stakeholders (e.g., community organizations, government/policy makers, hospital administration) to improve cancer care for youths?

The investigators plan to interview 8 each of youth cancer survivors, their parents/caregivers, and their HCP. A content analysis of semi-structured interviews will proceed prior to KM which includes the development of infographics, media interviews, publications, conference presentation, and an end-of grant forum.

Strengths and Weaknesses Noted Within Each Category on the Reviewer Grid.

Excellence of Researcher/Team:

- This team is commended for considering the pursuit of a qualitative study which could illuminate the experiences of youths who are cancer survivors, their parents/caregivers, and their HCP and identify care and service needs for this group.
- I note that this team includes expertise in pediatrics (including oncology, psychology, and palliative care) and qualitative methods. I would recommend the inclusion of an expert in knowledge translation, and survivor, patient and/or family carer.

Merit of the Proposal:

- Although the team makes the case that research of this kind has not often been carried out in a Canadian context (and not at all in the context of the Windsor-Essex region to my knowledge), the fact that similar studies have been conducted in related by distinct context (i.e., US, UK) somewhat diminishes the proposal's originality and innovative quality.
- Although the concept of the study is generally strong, the methodology is lacking some clarity and is only somewhat appropriately described. Some specific related points include:
    - It is not clear why only survivors are being recruited for this study. It may benefit the findings to include on-treatment patients who can provide immediate context related to their care experiences.

- The authors state youths will be “mainly” recruited from the POGO satellite clinical at Windsor Regional – is there another recruitment setting that should be listed.
- Many youth characteristics are included in the sampling matrix plan. It will be difficult to assign a youth to each cell of the matrix with the current sample size.
- There is discussion of recruiting youths based on race and ethnicity – but these items are not included in the matrix.
- How will family composition, gender identity, SES be determined for the purpose of recruitment?
- Should cancer diagnosis be considered in the maximum variation sampling procedure as it impact on cancer experience and may effect psychosocial and medical care needs?
- More details are required regarding the sample size. How was a sample size of 8 per group determined and will this be adequate to meet the needs of the study as designed?
- How was the semi-structured interview guide developed (what is its basis and who was involved in its design)?
- I am not sure, especially given the current sample sizes of 8 per group, that content analysis is the best analysis method. A thematic analysis may create a richer story with the data that can be used to highlight experiences and service needs in this area.
- Can details related to how rigor will be into the data collection and analysis methods be provided?

Impact:

- This research proposal has potential to lead to larger funding in this area in this important area of youth health and could support youth-, parent/caregiver-, and oncology HCP-directed advancements to clinical care in the Windsor-Essex Region.

WE-SPARK Igniting Discovery Grant Program
2023 Competition
Reviewers Comments

Project Title: Exploring the process of receiving pediatric cancer care in Windsor Essex Region through the lived experiences of youths living with cancer, parents, and healthcare providers.

Brief Overview of Proposal.

The proposed project seeks to understand gaps in medical and psychological care for youth who have received a cancer diagnosis, from the perspectives of youth, parents/caregivers, and health care professionals in Windsor, Ontario.

Strengths and Weaknesses Noted Within Each Category on the Reviewer Grid.

Excellence of Researcher/Team:

- Very robust research team with a combination of both academic and professional experience.
- The role of each team member is clearly defined.

Merit of the Proposal:

- The methodology is clearly defined.
- Background information is extensive.
- The proposal mentions inclusivity of all races and cultures; however, exclusion criteria for the study mentions those who do not speak English. This could result in the selected sample being less diverse. As such, it may be suggested that the research team explore methods/steps for still ensuring a diverse sample, such as recruitment strategies that demonstrate inclusivity.
- While the researchers have mentioned exploring the experiences of parents, healthcare providers, as well as patients, this may be a bit lofty for a robust analysis. It might be worthwhile to consider only involving parents and patients.

Impact:

- While the proposal itself does not appear entirely innovative, the project specifically concentrates on furthering pediatric cancer research from a Canadian lens. This is particularly relevant locally, as the grant mentions the out-of-city care that is required for those living in Windsor. This unique perspective could shed light on other, smaller communities that do not have the resources of larger urban centres in Canada.
- The proposal mentions the intersecting challenges associated with a cancer diagnosis in adolescence which is a unique factor that this project is seeking to explore.
- It would have added to the proposal to explore some of the more tangible potential advancements this research could lead to.
